# Future of Stroke Prevention: 7 Updates in the 2024 AHA/ASA Primary Prevention of Stroke Guideline

**DOI:** 10.1016/j.jacadv.2025.101724

**Published:** 2025-05-08

**Authors:** Alexander C. Razavi, Aaron L. Troy, Jaideep Patel, Laxmi S. Mehta, Jared A. Spitz, Donald Lloyd-Jones, Seamus P. Whelton, Michelle C. Johansen, Roger S. Blumenthal

**Affiliations:** a Emory Clinical Cardiovascular Research Institute, Emory University School of Medicine, Atlanta, Georgia, USA; b Ciccarone Center for the Prevention of Cardiovascular Disease, Johns Hopkins University School of Medicine, Baltimore, Maryland, USA; c Division of Cardiovascular Medicine, The Ohio State University Wexner Medical Center, Columbus, Ohio, USA; d Inova Schar Heart and Vascular Institute, Inova Health System, Fairfax, Virginia, USA; e Section of Preventive Medicine, Boston Medical Center, Boston, Massachusetts, USA; f Department of Neurology, Johns Hopkins University School of Medicine, Baltimore, Maryland, USA

**Keywords:** cardiovascular disease, cerebrovascular disease, primary prevention, stroke

## Abstract

Approximately 9 to 10 million adults (4%) have experienced a stroke in the United States. While stroke incidence has generally declined, progress has been less pronounced among young individuals, and such trends have underlined the importance of focusing on the primary prevention of stroke. In 2024, the American Heart Association and American Stroke Association released new guidelines for the primary prevention of stroke. Here, we review major updates in 7 domains: dietary modification, glucagon-like peptide-1 receptor agonists, blood pressure targets, lipid-lowering medications, antithrombotic agents, colchicine therapy, and sex-specific preventive risk assessment. Through this process, we review important randomized controlled trial evidence contributing to guideline updates and provide key perspectives on the incorporation of lifestyle and pharmacotherapy for personalized stroke prevention.

## Highlights

• Declines in stroke incidence have been less pronounced in younger adults.
• Benefits of <130/<80 mm Hg blood pressure extend to diabetes and older adults.
• New Class 1 indication for GLP-1RA in type 2 diabetes for stroke risk reduction.
• Further research required to define the precise role of colchicine in stroke risk reduction.

The prevalence of stroke in the United States is approximately 4% (9-10 million individuals), approaching 7% in persons ≥60 years old and 14% in those ≥80 years old.^1^ By 2050, the prevalence of stroke will likely have increased by nearly 66% when compared to 2020.^2^ While the incidence of stroke is generally declining in high-income countries,^3^ such progress is less pronounced among younger individuals—with stroke incidence rates remaining relatively flat or even increasing among those below age <55 years.^4^^,^^5^ Such trends underline the importance of focusing on the primary prevention of stroke among all age groups. Here we review 7 key updates to the recent^6^ American Heart Association/American Stroke Association 2024 Guideline on the Primary Prevention of Stroke.^6^

This 2024 Guideline embraces Life's Essential 8 for the promotion of cardiovascular disease (CVD) and brain health across the life course.^7^ In addition to emphasizing the importance of routine primary care and addressing adverse social determinants of health, this guideline featured major updates in 7 key domains for personalized stroke prevention: dietary modification, glucagon-like peptide-1 receptor agonists (GLP-1RA), blood pressure (BP) targets, lipid-lowering medications, antithrombotic agents, colchicine therapy, and sex-specific preventive considerations. In this review, we highlight advances in these key domains and the latest practice-changing literature behind the guidelines (Table 1, Central Illustration).

**Table 1:** 7 Key Summary Perspectives on the 2024 AHA/ASA Primary Prevention of Stroke Guideline

	Agent/Approach	2024 AHA/ASA Recommendation	LOE	Population	Perspectives
1. Dietary modification	Mediterranean diet	Class 1 (benefit)	B-randomized	Intermediate to high CVD risk	42% relative reduction of stroke (1.5% absolute risk reduction) in PREDIMED trial Whole grains, fruits, vegetables, beans, legumes, nuts, lean protein, olive oil
	Potassium salt substitution (75% NaCl, 25% KCl)	Class 2a (reasonable)	B-randomized	≥60 y old with SBP ≥140 mm Hg on BP medication or ≥160 mm Hg if no medication	22% relative reduction of stroke (0.34% absolute risk reduction) in SSaSS trial No data on incidence of hyperkalemia
2. Blood pressure target	BP target of <130/<80 mm Hg	Class 1 (benefit)	A	Stage 2 HTN or stage 1 HTN with high CVD risk	26% relative reduction in stroke (1.2% absolute risk reduction) for treatment to systolic blood pressure <130 vs ≥130 mm Hg Benefit extends to older adults (age ≥60 y) and those with type 2 diabetes
3. Lipid lowering	Statin	Class 1 (benefit)	A	Intermediate CVD risk and ≥1 risk enhancer High CVD risk T2D LDL-C ≥190 mg/dL	Similar statin eligibility groups as 2019 primary prevention of CVD guideline No discussion of statin therapy according to subclinical atherosclerosis burden Intermediate and high CVD risk groups likely to change with incorporation of PREVENT risk calculator
	PCSK9 mAb	Class 2b (uncertain)	A	Statin intolerant or those requiring further LDL-C lowering	21%-27% relative reduction in stroke for PCSK9 mAb vs placebo (<1% absolute risk reduction) Unclear whether benefit includes those without clinical CVD
	Bempedoic acid	Class 2b (uncertain)	B-randomized	Statin intolerant	Nonsignificant 15% relative reduction for stroke in CLEAR Outcomes trial CLEAR Outcomes was not powered to detect an independent effect on stroke
	Omega-3 FA	Class 3 (no benefit)	A	Adults with low to moderate omega-3 FA intake	Meta-analyses demonstrate that omega-3 FA do not reduce risk of stroke Significant heterogeneity in omega-3 FA trials in intervention and control groups
4. Antiplatelet and anticoagulation	Aspirin	Class 2b (uncertain)	A	T2D and/or traditional risk factors	Use of aspirin in those without clinical CVD is not well established Aspirin benefit groups may include those with advanced subclinical atherosclerosis, T2D, or elevated lipoprotein(a)
	Ticagrelor in DAPT (beyond 1 y, up to 3 y)	Class 2b (uncertain)	B-randomized	Stable CHD on aspirin therapy	15% relative reduction of stroke (0.47% absolute risk reduction) and 2.3-fold higher risk of major bleeding (1.2% absolute risk increase) among individuals with stable CHD on aspirin therapy randomized to receive 60 mg ticagrelor vs placebo
	Low-dose DOAC	Class 3 (harm)	B-randomized	LV systolic dysfunction (ejection fraction ≤35%-40%) without AF or LV thrombus	Evidence derived from COMMANDER HF trial
5. Risk reduction in T2D and obesity	GLP-1RA	Class 1 (benefit)	A	T2D	27% relative reduction (0.7% absolute risk reduction) in nonfatal stroke risk in T2D Evidence predominantly driven among those with established CVD No guidance provided for utilization in overweight or obese individuals
	Bariatric surgery	Class 2b (uncertain)	C-limited data	BMI ≥35-39 kg/m^2^	−34% to 51% relative reduction in stroke risk Data predominantly from case-control designs
	SGLT2i	No guidance provided			No evidence of significant stroke risk reduction in meta-analysis
6. Anti-inflammatory therapy	Low-dose colchicine	Class 2b (uncertain)	B-randomized	Adults with recent myocardial infarction on statin therapy	Signal for stroke prevention observed in COLCOT (74% relative reduction) Intention-to-treat analysis in CONVINCE: 21% relative reduction in recurrent stroke
7. Sex-specific risk assessment of stroke	Treatment of hypertension in pregnancy	Class 1 (benefit)	B-non randomized	Pregnant or postpartum women with BP ≥160/110 mm Hg	Targeting a goal BP <140/90 vs <160/105 mm Hg among pregnant women is associated with a 18% relative reduction in composite of preeclampsia with severe features, preterm birth, placental abruption, or fetal death (6.8% absolute risk reduction)
		Class 2a (reasonable)	C-limited data	Pregnant or postpartum women with BP ≥140/90 mm Hg	
	Screening for adverse pregnancy outcomes (APO)	Class 1 (benefit)	C-expert opinion	Pregnant women	APO defined by hypertension in pregnancy, preterm birth, gestational diabetes, and placental disorders Hypertension in pregnancy is the most common APO (13% to 15% of pregnancies) and associated with up to a 74% higher relative risk of stroke
	Screening for endometriosis	Class 2a (reasonable)	B-non randomized	Women	Approximate 16%-34% higher relative risk of stroke associated with endometriosis
	Screening for premature ovarian failure and early menopause	Class 1 (benefit)	B-non randomized	Women	Includes primary ovarian insufficiency, surgical oophorectomy, or medication-induced menopause Menopause <40 y old is associated with a 32% higher relative stroke risk
	Lowest possible estrogen-containing dose when considering contraception	Class 1 (benefit)	B-non randomized	Women	Screen for stroke specific risk factors in those considering contraception, including age >35 y old, tobacco use, hypertension, and migraine with aura

AF = atrial fibrillation; AHA = American Heart Association; ASA = American Stroke Association; BMI = body mass index; BP = blood pressure; CHD = coronary heart disease; CVD = cardiovascular disease; DAPT = dual antiplatelet therapy; DOAC = direct oral anticoagulant; FA = fatty acids; GLP-1RA = glucagon-like protein-1 receptor agonist; HTN = hypertension; KCl = potassium chloride; LDL-C = low-density lipoprotein-cholesterol; LOE = level of evidence; LV = left ventricular; mAb = monoclonal antibody; NaCl = sodium chloride; PCSK9 = proprotein convertase subtilisin/kexin type 9; SBP = systolic blood pressure; SGLT2i = sodium-glucose cotransporter 2 inhibitor; T2D = type 2 diabetes.

**Central Illustration:**
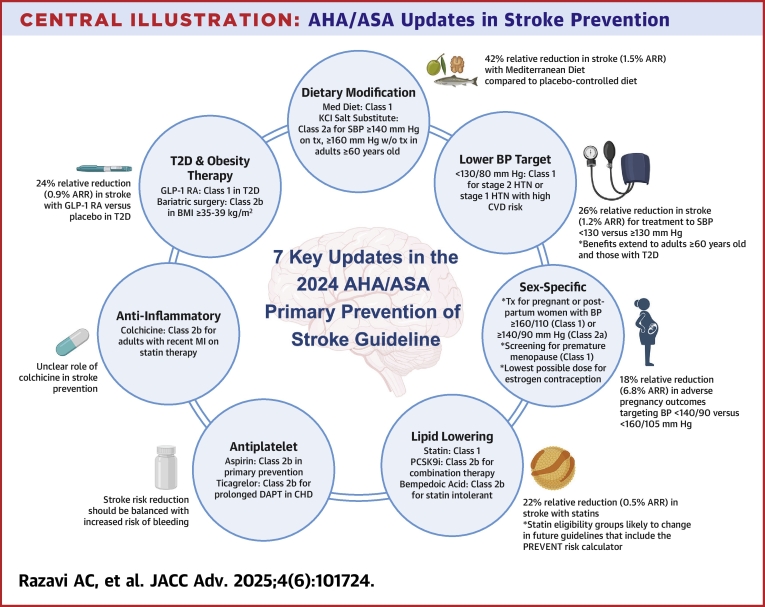
AHA/ASA Updates in Stroke Prevention Seven key updates in the 2024 AHA/ASA primary prevention of stroke guideline. AHA = American Heart Association; ARR = absolute risk reduction; ASA = American Stroke Association; BMI = body mass index; CHD = coronary heart disease; CVD = cardiovascular disease; GLP-1RA = glucagon-like protein-1 receptor agonist; HTN = hypertension; MI = myocardial infarction; SBP = systolic blood pressure; T2D = type 2 diabetes.

## Dietary modification

The latest guidelines underscore the preventive utility of the Mediterranean diet and salt substitution. With respect to nonpharmacological approaches for incident stroke risk reduction, the notable updates in the 2024 Guideline include a higher (Class 1, strong; indicating benefits substantially outweigh potential harms) recommendation for the Mediterranean diet and formal discussions involving potassium salt substitution. Among individuals with intermediate to high CVD risk, defined by the presence of 2 or more risk factors, a meta-analysis of randomized controlled trials (RCTs) indicates that Mediterranean diet interventions may reduce the risk of stroke by approximately 35% when compared to placebo-controlled diets.^8^ Importantly, Mediterranean dietary intervention was more strongly associated with stroke risk reduction (HR: 0.58; 95% CI: 0.42-0.82) when compared to myocardial infarction (HR: 0.80; 95% CI: 0.53-1.21) or CVD death (HR: 0.80; 95% CI: 0.51-1.24) in the PREDIMED (Prevention with Mediterranean Diet) trial.^9^

While the DASH (Dietary Approaches to Stop Hypertension) diet had a previous Class 1 indication in the 2014 Guideline due to RCT evidence for systolic BP reduction,^10^ no specific recommendations involving DASH were made in the 2024 update given the lack of RCT evidence including incident stroke as an outcome. Nevertheless, observational evidence suggests that each 4-point increase in DASH diet score is associated with a 4% relative risk reduction in stroke.^11^ Both the DASH and Mediterranean diets emphasize whole grains, fruits, and vegetables, though the Mediterranean diet has a higher average percentage of calories from monounsaturated (eg, olive oil) and polyunsaturated fat (eg, salmon, walnuts). In contrast to the Mediterranean and DASH diets, there is limited evidence that low fat dietary interventions result in significant risk reductions for stroke. Beyond the Mediterranean and DASH diets, observational evidence from the Nurses' Health Study suggests that adherence to a healthy plant-based diet may be associated with an 8% lower risk of ischemic stroke.^12^

The 2024 Guideline on the Primary Prevention of Stroke provided a new Class 2a (moderate; indicating benefits likely outweigh potential harms) recommendation for the use of salt substitutes (75% sodium chloride, 25% potassium chloride) rather than table salt (100% sodium chloride) to reduce stroke risk among adults ≥60 years of age with uncontrolled hypertension (systolic BP ≥140 mm Hg without medication, ≥160 on medication). This recommendation is based largely on a 22% relative risk reduction (1.17% vs 1.51%) in incident stroke among more than 5,000 individuals without prevalent stroke within a recent open-label, cluster RCT conducted in rural China.^13^ Additionally, results for the entire trial (individuals with and without stroke at baseline included) also demonstrated a significant 14% relative risk reduction in stroke with potassium salt substitution over 4.7 years. Notably, serum potassium levels were not measured in the primary RCT conducted involving salt substitution and clinicians should monitor closely for incident hyperkalemia among individuals on potassium-sparing diuretics, potassium supplementation, and/or those with chronic kidney disease (CKD).^7^

## GLP-1RA

GLP-1RA, such as semaglutide and dulaglutide, are recognized as a new class of medications for primary stroke prevention. Since 2019, utilization of GLP-1RA has increased by 7-fold in the United States.^14^ The 2024 Guideline provides a Class 1 (strong) recommendation for the utilization of GLP-1RA among individuals with a high predicted CVD risk or established CVD (including stroke) with a glycated hemoglobin value of ≥7% for primary and secondary stroke prevention. Meta-analyses of RCTs including GLP-1RA therapy among individuals with diabetes have indicated an approximate 27% reduction in nonfatal stroke risk—particularly driven by protection from ischemic stroke.^15^

Most individuals in RCTs involving GLP-1RA in type 2 diabetes have had prevalent CVD (73%-85%).^15^ The GLP-1RA outcomes trial in type 2 diabetes that included the most individuals *without* clinical CVD (∼70%) was REWIND (Research Cardiovascular Events with a Weekly Incretin in Diabetes).^16^ In the REWIND trial, <7% of participants had prevalent stroke at baseline and dulaglutide was associated with a 24% relative reduction in nonfatal stroke compared to placebo over a median 5.4-period follow-up (3.2% vs 4.1%; HR: 0.76; 95% CI: 0.62-0.94). There was no significant interaction for the protective association between dulaglutide and CVD according to baseline presence or absence of baseline clinical CVD.^16^ Further research is required to elucidate whether GLP-1RA therapy in those without type 2 diabetes with overweight or obesity reduces the risk of incident or recurrent stroke.^16^ Beyond GLP-1RA, meta-analysis of RCTs has not found that sodium-glucose cotransporter-2 inhibitors therapy significantly reduces the risk of stroke among individuals who have type 2 diabetes with or without clinical CVD.^17^

## BP targets

Similar to recent major societal guidelines,^18^^,^^19^ the 2024 Guideline for the Primary Prevention of Stroke provides a Class 1 (strong) recommendation for targeting a new (lower compared to the 2014 stroke guideline) systolic BP target of <130 and diastolic BP <80 mm Hg in adults at higher risk for atherosclerotic CVD.^6^ While higher risk for atherosclerotic CVD was not formally defined in the 2024 Guideline, it may be inferred based on RCT evidence that this population is generally reflective of individuals with clinical CVD, advanced subclinical atherosclerosis, CKD, type 2 diabetes, older adults, or a high 10-year risk of CVD. Rigorous evidence has demonstrated a benefit of targeting a systolic BP of <130 and diastolic BP <80 mm Hg for the primary and secondary prevention of stroke,^20^ including older adults ≥60 years of age and those with type 2 diabetes. Each 5 mm Hg reduction in systolic BP reduction confers an approximate 13% lower risk of stroke among individuals with and without clinical CVD.

In the STEP (Strategy of Blood Pressure Intervention in the Elderly Hypertensive Patients) trial,^21^ individuals randomized to intensive vs standard antihypertensive treatment (<130 vs <150 mm Hg) experienced a 33% lower risk of incident stroke over 1 year of follow-up (0.3% vs 0.5%). Among patients with diabetes, the ACCORD (Action to Control Cardiovascular Risk in Diabetes) BP trial targeted a systolic BP <120 vs <140 mm Hg and observed a 41% lower risk of stroke over 1 year (0.3% vs 0.5%), which was a secondary outcome of the trial.^22^ While there was no significant risk reduction observed for the primary outcome of major adverse cardiovascular events in ACCORD, this may have been due to the factorial design (BP and glucose-lowering) of the ACCORD trial.

More recently, after the 2024 Guideline's publication, the BPROAD (Blood Pressure Control Target in Diabetes) trial demonstrated that intensive treatment to a systolic BP of ≤120 vs ≤140 mm Hg among more than 12,000 individuals with type 2 diabetes resulted in a 21% relative risk reduction in stroke (1.65% vs 2.09%) over 5 years.^23^ Importantly, this reduction in stroke appeared to be largely responsible for the reduction in the primary composite CVD outcome. One meta-analysis has demonstrated a 26% relative reduction in stroke (HR: 0.74; 95% CI: 0.66-0.84; 3.3% vs 4.5%) among 7 RCTs assessing antihypertensive targeting a systolic BP of <130 vs ≥130 mm Hg, and a 19% relative reduction in stroke (HR: 0.81; 95% CI: 0.70-0.94; 3.1% vs 3.7%) among 4 RCTs targeting a systolic BP of <120 vs ≤140 mm Hg.^24^ Based on RCT evidence, the 2024 European Society of Cardiology Guideline for the Management of Elevated Blood Pressure and Hypertension provide a Class 1 recommendation to target a systolic BP treatment target of 120 to 129 mm Hg if therapy is well-tolerated.^25^

## Lipid-lowering therapy

Similar to the 2019 American College of Cardiology/American Heart Association Primary Prevention of CVD Guideline,^18^ the 2024 Primary Prevention of Stroke Guideline recommends (Class 1) primary prevention statin therapy across 4 statin eligibility groups (low-density lipoprotein-cholesterol [LDL-C] ≥190 mg/dL, type 2 diabetes, 10-year CVD risk ≥20%, and 10-year CVD risk between 7.5% and 19.9% with at least one risk enhancer).^18^^,^^26^ However, the classification for intermediate and high risk may change when guideline updates use the 2023 PREVENT (Predicting Risk of Cardiovascular Disease Events) calculator,^27^^,^^28^ which generally provides a risk estimate 30% to 50% lower compared to the 2013 Pooled Cohort Equations owing to its derivation in a much larger and more contemporary sample.

Utilization of proprotein convertase subtilisin/kexin type 9 (PCSK9) monoclonal antibodies (mAbs) for statin-eligible individuals who are statin intolerant or require further LDL-C lowering on maximally tolerated statin therapy was given a 2b recommendation (uncertain benefit). This recommendation is made based on the fact that: 1) no dedicated primary prevention trials involving PCSK9 mAbs (evolocumab, alirocumab) have been performed; and 2) one meta-analysis identified 21% to 27% risk reduction (95% CI: 6%-42%) in stroke for PCSK9 mAbs when compared to placebo but did not specify whether individuals had prevalent stroke at baseline, thereby making recommendations for primary prevention of stroke unclear.^29^

In the ODYSSEY OUTCOMES (Evaluation of Cardiovascular Outcomes After an Acute Coronary Syndrome During Treatment with Alirocumab) trial, alirocumab conferred a lower risk of fatal and nonfatal stroke in secondary outcome analysis (27% relative risk reduction, 1.2% vs 1.6%) over nearly 3 years among persons with acute coronary syndrome within the last 12 months.^30^ Approximately 3% of individuals in ODYSSEY OUTCOMES had prevalent stroke and all were on maximally tolerated statin therapy. However, evidence from the FOURIER (Further Cardiovascular Outcomes Research with PCSK9 Inhibition in Subjects with Elevated Risk) trial did not show a significant risk reduction in stroke with evolocumab within secondary outcome analysis.^31^^,^^32^

New recommendations for the utilization of bempedoic acid and omega-3 fatty acid supplementation/pharmacotherapy were provided in the 2024 Primary Prevention of Stroke Guideline. Use of bempedoic acid among individuals with statin-associated side effects received a 2b recommendation in the 2024 Primary Prevention of Stroke Guideline. In secondary RCT outcomes, bempedoic acid led to a nonsignificant 15% relative risk reduction (HR: 0.85; 95% CI: 0.67-1.07) in fatal or nonfatal stroke; however, the CLEAR Outcomes trial was not powered to detect an independent effect on stroke.^33^

A Class 3 recommendation (no benefit) was provided for omega-3 fatty acid supplementation for stroke risk reduction. Several different meta-analyses have demonstrated that omega-3 fatty acid supplementation does not reduce risk of stroke.^34^^,^^35^ However, not all RCTs involving omega-3 fatty acids included in these meta-analyses have included similar control groups^36^^,^^37^ or similar omega-3 fatty acid formulations, which may be an important consideration given that high-dose eicosapentaenoic acid and icosapent ethyl monotherapy may have enhanced CVD protective benefit compared to combinations including docosahexaenoic acid.^38^ REDUCE-IT (Reduction of Cardiovascular Events with Icosapent Ethyl–Intervention Trial) found that 2 g of twice daily icosapent ethyl vs mineral oil placebo led to a 28% relative risk reduction in a secondary outcome of stroke (2.4% vs 3.3%),^39^ although subgroup analyses were not reported according to the presence vs absence of baseline stroke and/or prevalent clinical CVD. Providers should remain focused on optimal statin therapy, with the consideration of PCSK9 mAb for further LDL-C lowering or bempedoic acid in those with statin intolerance.

## Antiplatelet and anticoagulation therapies

The 2024 Guideline on the Primary Prevention of Stroke emphasizes the judicious and intentional use of antithrombotic and anticoagulant agents for stroke prevention. In contrast to the 2014 Stroke Prevention Guideline that provided a Class 2a recommendation (reasonable) for the utilization of aspirin therapy among individuals without clinical CVD who had a 10-year risk ≥10% or those with diabetes,^26^ the 2019 Primary Prevention of CVD Guideline^18^ provided a 2b recommendation (uncertain) for the utilization of aspirin therapy in those without prior CVD, including diabetes and/or other traditional risk factors.^6^ While observational evidence suggests that individuals with advanced subclinical atherosclerosis^40^, ^41^, ^42^ and/or elevated lipoprotein(a)^43^, ^44^, ^45^ may derive net benefit (CVD risk reduction benefit outweighing risk of major bleeding) from primary prevention aspirin therapy, there are limited data specific for the primary prevention of stroke unlike for coronary heart disease (CHD). Class 3 recommendations (harm) for primary prevention aspirin therapy are provided for individuals with CKD and those ≥70 years of age without atherosclerotic CVD.

Newly added to the 2024 Guideline is a 2b recommendation for the use of ticagrelor as a dual antiplatelet agent beyond 12 months, and up to 3 years, in addition to aspirin, for the prevention of ischemic stroke in those with stable CHD with low bleeding risk. This recommendation is derived from the PEGASUS-TIMI 54 (Prevention of Cardiovascular Events in Patients with Prior Heart Attack Using Ticagrelor Compared to Placebo on a Background of Aspirin-Thrombolysis In Myocardial Infarction^54^) trial, where individuals with a history of myocardial infarction on background aspirin therapy randomized to receive 60 mg ticagrelor vs placebo experienced a 15% relative risk reduction of stroke (1.47% vs 1.94%).^46^ However, there was at least a 2.3-fold higher risk of major bleeding events (2.3% vs 1.1%) and a 2.8-fold higher risk of dyspnea (15.8% vs 6.4%) among individuals randomized to receive ticagrelor vs placebo.

Additionally, recommendations regarding aspirin and/or direct oral anticoagulant therapy for individuals with decreased left ventricular (LV) systolic function (ejection fraction ≤35%-40%) and no atrial fibrillation (AF) or LV thrombus are provided. Based on evidence from the WARCEF (Warfarin vs Aspirin in Reduced Cardiac Ejection Fraction)^47^ and COMMANDER HF (A Study to Assess the Effectiveness and Safety of Rivaroxaban in Reducing the Risk of Death, Myocardial Infarction or Stroke in Participants with Heart Failure and Coronary Artery Disease Following an Episode of Decompensated Heart Failure)^48^ trials, the 2024 Guideline does not recommend the use of anticoagulation among individuals with a reduced LV ejection fraction and no AF or LV thrombus due to an increased risk of major bleeding events (Class 3 recommendation, harm).

Although not directly addressed in the 2024 Primary Prevention of Stroke Guideline, anticoagulation for primary prevention of thromboembolic stroke among individuals with AF is covered in the 2023 Diagnosis and Management of Atrial Fibrillation Guideline. Among individuals with AF who have an annual risk of thromboembolic stroke ≥2% (CHA_2_DS_2_-VASc ≥2 in men or ≥3 in women), selection of anticoagulation is recommended to be based upon the annualized stroke rather than the pattern of AF (paroxysmal, persistent, long-standing persistent, or permanent).^49^

It is important for clinicians to have shared decision-making conversations with patients about their individualized stroke and bleeding risk, particularly for patients at very high stroke risk but whom have no other clear indication for antiplatelet or anticoagulant therapy.

## Colchicine

Colchicine is an anti-inflammatory agent that has wide-ranging properties, ranging from tubulin polymerization inhibition and moderation of leukocyte activity.^50^ The 2024 Primary Prevention of Stroke Guideline provided a 2b recommendation (moderate) for the utilization of low-dose colchicine among individuals with a recent myocardial infarction, defined as 30 days from the myocardial infarction. There have been several RCTs performed to assess the role of colchicine in CVD risk reduction since 2014, including LoDoCo (Low-Dose Colchine),^51^ COLCOT (Colchicine Cardiovascular Outcomes Trial),^52^ LoDoCo2 (Low-Dose Colchicine 2),^53^ and most recently CLEAR SYNERGY OASIS 9 (Colchicine in Acute Myocardial Infarction). All RCTs included individuals with stable CHD.

While the COLCOT^52^ trial identified a 74% relative risk reduction in a planned secondary endpoint of stroke among individuals randomized to receive 0.5 mg colchicine vs placebo, the LoDoCo^51^ (HR: 0.23; 95% CI: 0.03-2.03) and LoDoCo2 (HR: 0.66; 95% CI: 0.35-1.25) trials did not observe significant risk reductions for stroke among individuals with stable CHD—although the latter trials were not adequately powered for stroke. The largest RCT involving colchicine among individuals with CHD (CLEAR SYNERGY-OASIS 9) did not observe any significant reduction in a composite primary CVD outcome including recurrent myocardial infarction, stroke, or ischemia-driven coronary revascularization (HR: 0.99; 95% CI: 0.85-1.16) or secondary outcome including stroke alone (HR: 1.15; 95% CI: 0.72-1.84) for persons randomized to 0.5 mg colchicine vs placebo.

Beyond individuals with stable CHD, the CONVINCE (Colchicine for preventioN of Vascular Inflammation in No-CardioEmbolic stroke) trial was conducted to assess the role of colchicine for preventing recurrent vascular events among individuals with prevalent stroke over 3 years.^54^ The intention-to-treat analysis in this trial did not meet statistical significance; however, per-protocol analysis demonstrated a 21% relative risk reduction in CVD with 0.5 mg colchicine vs placebo (3.1% vs 3.8%), which appeared to be driven by prevention of recurrent stroke.

Currently, it is unclear whether low-dose colchicine significantly lowers the risk of stroke; however, low-dose colchicine may be considered among individuals stable CHD with normal kidney function.

## Sex-specific risk assessment for the primary prevention of stroke

In 2019, more than one-half (57%) of all stroke deaths were among women,^55^ as women have a slightly higher lifetime risk of stroke compared to men (20%-27% vs 14%-17%).^56^ The 2024 Primary Prevention of Stroke Guideline dedicated a new section for sex-specific risk assessment and management. The guideline emphasized the prognostic and therapeutic implications of pregnancy-associated stroke, screening for endometriosis, premature ovarian failure, and early onset menopause, as well as care of individuals on sex hormone supplementation.

For the prevention of pregnancy-related stroke, the 2024 Guideline provided recommendations for the management of hypertensive disorders of pregnancy. Among women with severe hypertension in pregnancy or postpartum, defined by a systolic BP ≥160 or diastolic BP ≥110 mm Hg on 2 separate measurements, a Class 1 recommendation was provided for immediate antihypertensive treatment for the prevention of maternal intracerebral hemorrhage.^57^ A Class 2a recommendation was provided to target a goal BP of <140/90 mm Hg for all women, with either chronic hypertension before or during pregnancy or who develop hypertensive disorders of pregnancy, specifically to reduce the risk of pregnancy-associated stroke.

The guidelines strongly recommend providers routinely screen for premature ovarian failure (before 40 years of age) and early-onset menopause (before 45 years of age) (Class 1) during clinical visits for the prevention of stroke. To mitigate CVD risk, the most optimal candidates for hormone therapy for the moderation of vasomotor symptoms in premature ovarian failure or early-onset menopause include those <60 years old who have experienced menopause within the last 10 years and do not have elevated risk for CVD, stroke, or breast cancer.^6^ Estrogen therapy among transgender women is also associated with a significantly higher risk of stroke,^58^ and a 2a recommendation is provided for risk factor evaluation and modification in this patient population.

## Conclusions

The 2024 Guideline on the Primary Prevention of Stroke provides important updates and new considerations for the primary prevention of stroke, based on the latest available evidence. Healthy lifestyle, including adherence to a Mediterranean dietary pattern, regular physical activity, adequate sleep, and traditional risk factor control remain the foundation for the primary prevention of stroke. Beyond statin therapy, the 2024 Primary Prevention of Stroke Guideline provides important guidance for the potential earlier incorporation of residual risk-lowering therapies, most strongly for the utilization of GLP-1RA in those with type 2 diabetes for stroke risk reduction. Ongoing studies will be required to guide the precise role of combination lipid-lowering therapy and colchicine for potential stroke risk reduction in those that derive maximal net benefit. Beyond lifestyle and pharmacotherapy, clinicians should strive to incorporate sex-specific risk assessment, including screening for premature ovarian failure and early-onset menopause for stroke prevention in women, and more intensive BP management in pregnancy.

## Funding support and author disclosures

Dr Razavi is supported by the National Heart, Lung, and Blood Institute Grants F32HL172499 and L30HL175751. All other authors have reported that they have no relationships relevant to the contents of this paper to disclose.
